# 8-Year Outcomes After Sleeve Gastrectomy: Anthropometric, Biochemical, and Nutritional Evaluation

**DOI:** 10.1007/s11695-026-08689-z

**Published:** 2026-04-27

**Authors:** Seher Şen

**Affiliations:** Department of Nutrition and Dietetics, Faculty of Health Sciences, Mudanya University, Bursa, Turkey

**Keywords:** Sleeve gastrectomy, Recurrent weight gain, Long-term outcomes, Dietary patterns, Metabolic outcome

## Abstract

**Introduction:**

Comprehensive long-term studies simultaneously evaluating anthropometric, biochemical, and nutritional outcomes after sleeve gastrectomy (SG) remain limited. This study aimed to compare preoperative findings with outcomes at postoperative year 8 and to explore factors associated with recurrent weight gain (RWG).

**Methods:**

This longitudinal observational study involved the prospective reassessment of a retrospectively defined cohort of patients who underwent SG between 2016 and 2017. Anthropometric measurements, biochemical parameters, dietary intake, lifestyle behaviors, and associated medical conditions were evaluated both preoperatively and at the 8-year postoperative follow-up. RWG and suboptimal clinical response were defined according to IFSO consensus criteria. Nutritional intake was assessed using 3-day dietary records, and statistical analyses were conducted using non-parametric methods.

**Results:**

Reductions were observed in body weight, waist circumference, waist-to-hip ratio, and body fat percentage (*p* < 0.05). Improvements in cardiometabolic parameters were also observed, including lower fasting glucose and HOMA-IR levels and more favorable lipid profiles. RWG was identified in 52.2% ofpatients when assessed using current weight. Higher energy intake and less favorable dietary patterns, including increased refined carbohydrate intake, were observed among patients with RWG. Differences in meal timing and adherence to postoperative eating recommendations were also observed between groups. Despite attenuation of peak weight loss over time, metabolic benefits were largely maintained.

**Conclusion:**

Long-term outcomes after SG involve both initial weight loss and sustained behavioral adaptation. RWG was observed in a substantial proportion of patients; however, overall weight loss and metabolic improvements were largely preserved. Given the exploratory nature and limited sample size, findings should be interpreted with caution, and further prospective studies are needed.

## Introduction

Obesity continues to represent a major global public health challenge, with its prevalence steadily increasing worldwide [1]. Metabolic Bariatric Surgery (MBS) is currently considered the most effective therapeutic approach for patients with severe obesity (BMI ≥ 35 kg/m²), providing sustained long-term weight reduction and improvement in associated medical conditions [2–4]. However, recurrent weight gain (RWG) is recognized as a common long-term concern following bariatric procedures, typically observed between the second and tenth postoperative years [5–9].

Studies report that 11–22% of patients experience RWG within the first 1–2 years following MBS [10–13]. With longer follow-up, higher rates of RWG have been reported, reaching up to 64% and 76% at postoperative years 4 and 6, respectively [6, 12].

SG, one of the most frequently performed bariatric procedures worldwide, has also been associated with variable long-term weight trajectories. RWG after SG has been reported in 5.7% of patients at 2 years and in up to 75.6% at 6 years postoperatively [6, 7, 14–16]. In contrast, a retrospective study by Iossa et al. reported sustained long-term weight reduction over a 7-year period following SG [17]. These findings suggest that long-term outcomes after SG vary considerably between patients, with some experiencing RWG or a suboptimal clinical response, which may lead to consideration of conversion bariatric surgery (CBS) [18].

Most studies evaluating long-term outcomes after SG primarily focus on weight-related outcomes, surgical complications, and associated medical conditions [8, 19]. In contrast, comprehensive long-term investigations that simultaneously assess anthropometric measurements, biochemical parameters, and nutritional status remain limited. A broader evaluation of these domains is essential to better understand the sustainability of SG and its long-term metabolic implications.

The aim of the present study was to compare preoperative findings with outcomes at postoperative year 8 in patients who underwent SG between 2016 and 2017. Anthropometric measurements, biochemical parameters, and nutritional status were reassessed to evaluate long-term outcomes and to provide extended follow-up data in this population.

## Methods

### Study Design and Participant Selection

This study is a longitudinal observational investigation conducted through the prospective reassessment of a retrospectively defined cohort. The study included patients who underwent SG at a private hospital between September 2016 and June 2017.

**Fig. 1:**
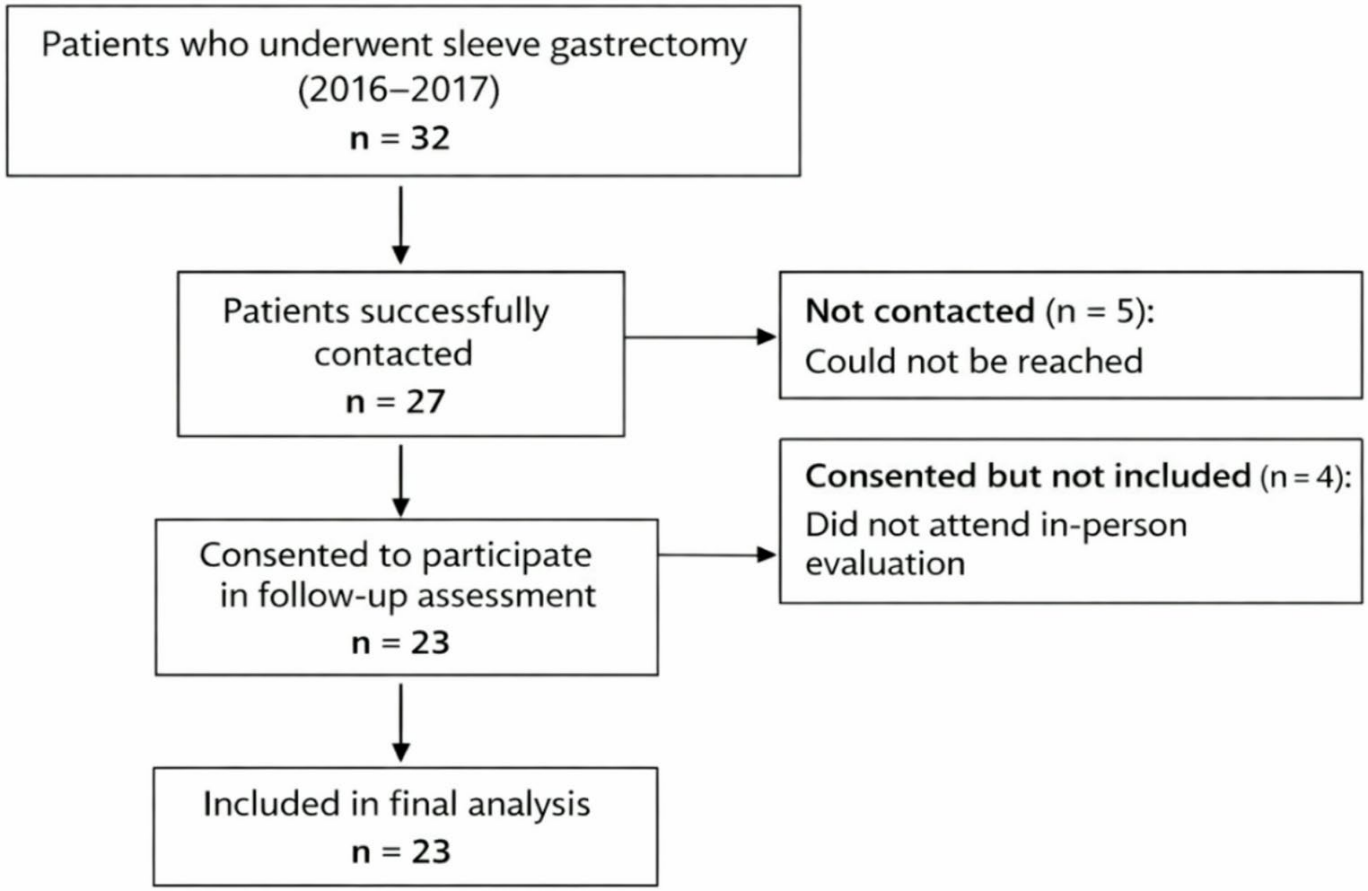
STROBE flow diagram of patient selection and 8-year follow-up after SG

A total of 32 patients underwent SG during the study period. Of these, 27 patients were successfully contacted for follow-up, and 23 agreed to participate in the 8-year reassessment and were included in the final analysis. Four patients who consented to participate were excluded due to non-attendance at the scheduled in-person evaluation. A flow diagram illustrating participant selection has been provided (Fig. 1).

Preoperative anthropometric measurements, biochemical parameters, and nutritional assessments were obtained from medical records.

In the second phase of the study, the same patients were re-invited for reassessment at the 8-year postoperative follow-up. Anthropometric measurements, biochemical parameters, and nutritional status were re-evaluated. This design enabled direct comparison between preoperative baseline findings and outcomes at postoperative year 8.

The sample size was determined by the number of eligible patients available for long-term follow-up. Given the observational design, no formal sample size calculation was performed for subgroup or multivariable analyses, and the study should be considered exploratory.

Inclusion criteria were patients aged 18–75 years who had undergone SG 8 years earlier and who had documented preoperative anthropometric measurements, biochemical findings, and nutritional assessments.

Exclusion criteria included pregnancy or lactation, the presence of acute illness or infection, associated medical conditions that could limit treatment safety or evaluation, and participation in professional athletic activity [16].

### Study Approval

All patients received both verbal and written information regarding the study procedures, and written informed consent was obtained prior to enrollment.

The study was conducted in accordance with the principles of the Declaration of Helsinki. The study protocol was reviewed and approved by a Non-Interventional Clinical Research Ethics Committee (Approval No: 838; Protocol No: E-10840098-202.3.02-4843; Date: July 17, 2025). The study was also registered at ClinicalTrials.gov (Identifier: NCT07348822).

### Dietary Interventions

Individuals were followed regularly by a registered dietitian during the first 6 postoperative months. A structured four-stage nutritional program consistent with the recommendations of the American Society for Metabolic and Bariatric Surgery (ASMBS) was implemented. At postoperative month 6, patients received education focused on sustainable long-term dietary practices.

### Study Outcomes and Measurements

Anthropometric and biochemical parameters were assessed preoperatively and at postoperative year 8. Evaluated anthropometric variables included body weight, body mass index (BMI), waist circumference, hip circumference, waist-to-hip ratio (WHR), body fat percentage, percentage of total weight loss (%TWL), and percentage of excess weight loss (%EWL).

Body fat percentage was measured using bioelectrical impedance analysis (BIA) (Tanita MC-780 MA, Japan) in accordance with ESPEN guidelines [20]. Measurements were performed under standardized conditions with patients wearing light clothing and barefoot. Patients were instructed to fast for at least 4 h prior to measurement and to avoid alcohol, caffeine, and exercise for at least 8 h. All metallic objects (e.g., jewelry, watches) were removed before analysis [21].

%TWL was calculated as:

$$\:\frac{(\text{Preoperative weight}-\text{Postoperative weight})}{\text{Preoperative weight}}\times\:100$$

%TWL was categorized as moderate (20–25%) or high (≥ 25%) [6, 22, 23].

%EWL was calculated as:

$$\:\:\frac{(\text{Preoperative weight}-\text{Postoperative weight})}{(\text{Preoperative weight}-\text{Ideal weight})}\times\:100$$

Ideal body weight was defined as the weight corresponding to a BMI of 25 kg/m² [24].

RWG was defined according to IFSO consensus recommendations. Patients were categorized based on a cut-off value of 30% weight regain from maximum weight loss (≥ 30% vs. < 30%). Relative RWG was calculated as: [25].

At baseline and postoperative year 8, face-to-face interviews were conducted to collect food frequency data and 3-day dietary records, including one weekend day. Daily macro- and micronutrient intake was analyzed using a computer-assisted nutrition analysis software program (Nutrition Information Systems – BEBIS 9, Version 9.0, Istanbul, Türkiye) [26].

Biochemical evaluation included fasting plasma glucose, HbA1c, HDL, LDL, total cholesterol, triglycerides, ALT, AST, urea, albumin, hemoglobin, hematocrit, ferritin, serum iron, vitamin B12, folate, total calcium, sodium, and potassium levels. Patients were instructed to fast for at least 8 h before blood sampling.

Additionally, physician-diagnosed medical conditions at the preoperative stage and at postoperative year 8 were recorded, including patients receiving medical treatment as well as those managed through lifestyle interventions. Daily step counts and habitual sleep duration were also assessed.

### Statistical Analysis

Normality of continuous variables was evaluated using the Shapiro–Wilk test. As the data were not normally distributed, non-parametric statistical methods were applied. Paired categorical variables were compared using the exact two-sided McNemar test. Within-group comparisons of paired continuous variables were performed using the Wilcoxon signed-rank test. Between-group comparisons were conducted using the Mann–Whitney U test for continuous variables and the chi-square test or Fisher’s exact test for categorical variables, as appropriate. All statistical analyses were conducted using IBM SPSS Statistics version 25.0 (IBM Corp., Armonk, NY, USA). A p-value < 0.05 was considered statistically significant. 

## Results

A total of 32 patients underwent sleeve gastrectomy between 2016 and 2017. Of these, 27 patients were successfully contacted for follow-up. Twenty-three patients agreed to participate in the 8-year reassessment and were included in the final analysis. Five patients could not be reached, and four patients were excluded due to non-attendance at the scheduled in-person evaluation. A STROBE-compliant flow diagram illustrating participant selection and follow-up is presented in Fig. 1.

Baseline characteristics of patients who participated in the 8-year reassessment and those who did not are presented in Table 1.

**Table 1:** Comparison of baseline characteristics between participating and non-participating patients

Variable	Participating(*n* = 23)	Non-participating(*n* = 9)	*p*
Age (years)	47.9 ± 11.8	39.3 ± 7.8	0.056
Sex (female, n %)	19 (82.6%)	7 (77.8%)	1.000
Preoperative weight (kg)	127.2 ± 24.4	129.7 ± 14.7	0.779
Preoperative BMI (kg/m²)	45.7 ± 11.4	48.7 ± 3.5	0.451

Mean ± SD or n (%); independent t-test and Fisher’s exact test

**Fig. 2:**
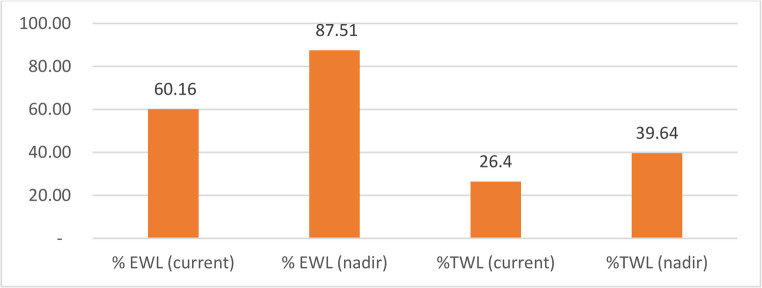
Comparison of mean %EWL and %TWL calculated using current versus nadir weight during the 8-year period

**Fig. 3:**
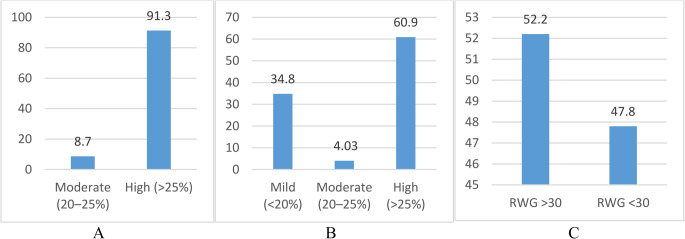
Comparison of %TWL and %RWG categories based on current versus nadir weight over an 8-year period

Patients’ demographic characteristics are presented in Table 2, and changes in associated medical conditions between the preoperative period and postoperative year 8 are shown in Table 3. The comparison of mean %EWL and %TWL calculated using current and nadir body weight is illustrated in Fig. 2. The distribution of patients according to %TWL and RWG categories is presented in Fig. 3.

**Table 2:** Patients’ demographic characteristics

Variable	Value
Age (years)	47 (36–52)
Sex, n (%)	
Female	19 (82.6)
Male	4 (17.4)
Education level, n (%)	
Middle school	6 (26.1)
High school	6 (26.1)
University	11 (47.8)
Employment status, n (%)	
Employed	13 (56.5)
Unemployed	10 (43.5)

Independent samples t-test and Fisher’s exact test were used

**Table 3:** Changes in comorbidities from preoperative period to postoperative year 8 (*n* = 23)

Comorbidity	Preop *n* (%)	Postop 8. years *n* (%)	*p* *
Diabetes mellitus	4 (17.4)	2 (8.7)	0.500
Insulin resistance	22 (95.7)	8 (34.8)	< 0.001
Hyperlipidemia	5 (21.7)	1 (4.3)	0.125
Hypertension	8 (34.8)	5 (21.7)	0.375
Obstructive sleep apnea	11 (47.8)	0 (0.0)	< 0.001
Asthma	3 (13.0)	2 (8.7)	1.000
GERD	15 (65.2)	9 (39.1)	0.180
FMF	2 (8.7)	2 (8.7)	1.000
Hypothyroidism	3 (13.0)	3 (13.0)	1.000
PCOS	9 (39.1)	1 (4.3)	0.008

*GERD* Gastroesophageal Reflux Disease, *FMF* Familial Mediterranean Fever, *PCOS* Polycystic Ovary Syndrome, *Preop* preoperative, *Postop *postoperative

*McNemar test (exact, two-sided)

**Fig. 4:**
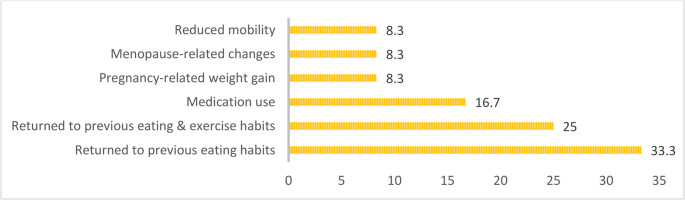
Post-regain factors among patients with > 30% RWG during the 8-year period (*n* = 12)

**Fig. 5:**
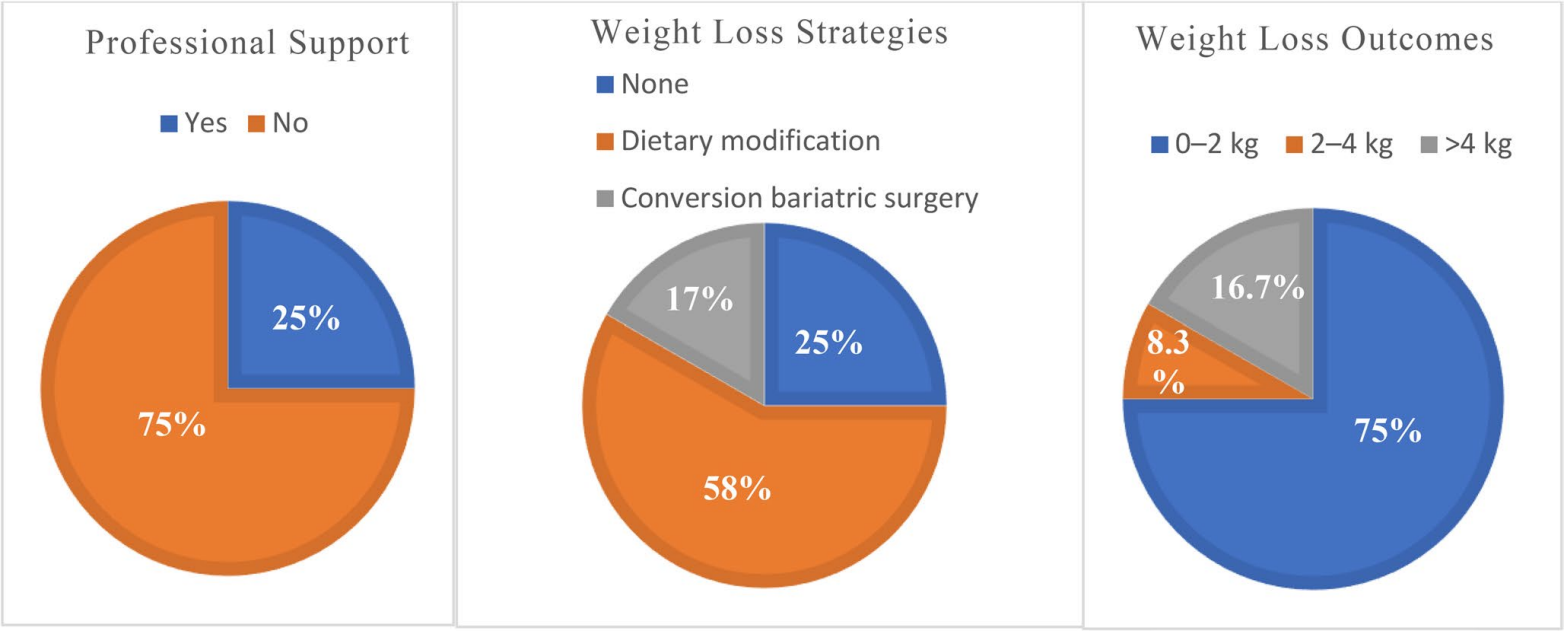
Professional support, weight loss strategies, and weight loss outcomes in patients with RWG

Among patients with RWG, reported reasons for weight regain were evaluated. In patients with RWG ≥ 30% (*n* = 12), the most frequently reported reason was a return to previous eating behaviors (33.3%) (Fig. 4). 75% of patients with RWG reported not receiving professional support. The most commonly used weight management strategy was dietary modification (58%), whereas 25% reported not using any method and 17% underwent CBS. Weight reduction achieved during attempts to lose weight again was 0–2 kg in 75% of patients, 2–4 kg in 8.3%, and ≥ 4 kg in 16.7% (Fig. 5).

Reductions were observed in all anthropometric measurements between the preoperative period and postoperative year 8 (*p* < 0.05) (Table 4). No difference was observed between %TWL categories and RWG status (Fisher’s exact test, *p* = 1.000) (Table 5).

**Table 4:** Anthropometric measurements of patients preoperatively and at postoperative year 8

	Preop Median (min-max)	Postop 8. years Median(min-max)	p*
Weight (kg)	127.8 (87.0–182.0)	88.0 (59.6–124.8)	< 0.001
BMI (kg/m²)	47.0 (30.9–62.6)	33.1 (23.3–43.5)	< 0.001
WC (cm)	126 (102–164)	97 (79–140)	< 0.001
HC (cm)	140.0 (118.0–187.0)	118.0 (98.7–145.0)	< 0.001
MHR	0.92 (0.70–1.15)	0.86 (0.66–1.03)	< 0.003
WtHR	0.79 (0.61–1.01)	0.63 (0.48–0.87)	< 0.001
Body fat (%)	53.00 (40.68–60.50)	38.67 (23.30–56.00)	< 0.001

*BMI* Body Mass Index, *WC* Waist Circumference, *HC* Hip Circumference, *WHR* Waist-to-Hip Ratio, *WHtR *Waist-to-Height Ratio, *Preop* preoperative, *Postop* postoperative

*Wilcoxon signed-rank test

**Table 5:** Comparison between %TWL categories and RWG at postoperative year 8

%TWL category	RWG > 30%	RWG < 30%	Total
Moderate (20–25%)	1 (50.0%)	1 (50.0%)	2 (100%)
High (> 25%)	11 (52.4%)	10 (47.6%)	21 (100%)
Total	12 (52.2%)	11 (47.8%)	23 (100%)

*Fisher’s exact test (two-sided), *p* = 1.000

Biochemical parameters and dietary intake comparisons between the preoperative period and postoperative year 8 are presented in Tables 6 and 7. Postoperative year-8 macronutrient intake according to RWG status is summarized in Table 8. Differences in eating habits and lifestyle behaviors according to RWG status are shown in Table 9. Preoperative and postoperative dietary patterns of patients with RWG are presented in Table 10.

**Table 6:** Comparison of biochemical parameters between the preoperative and postoperative year 8

	Preop median(Min-Max)	Postop median(Min-Max)	P*
FPG (mg/dL)	101.5 (85.2–174.0)	93.0 (67.0–108.0)	0.021
HOMA-IR	5.10 (2.50–8.90)	2.47 (1.28–7.10)	**< **0.001
Hemoglobin A1c (%)	5.43 (5.04–8.50)	5.50 (5.10–6.19)	0.879
Urea (mg/dL)	26.0 (13.0–49.0)	20.0 (13.0–28.0)	0.003
Albumin (g/dL)	4.30 (3.50–5.00)	4.20 (3.84–5.10)	0.262
HDL-C (mg/dL)	44.0 (35.0–61.1)	57.0 (26.0–101.0)	0.005
LDL-C (mg/dL)	127.8 (85.0–182.2)	112.0 (62.0–166.0)	0.019
Total Cholesterol (mg/dL)	214.0 (142.0–267.0)	189.0 (138.0–264.0)	0.494
Triglyceride (mg/dL)	122.0 (56.0–316.0)	122.0 (56.0–172.0)	0.052
ALT (U/L)	23.8 (9.1–98.3)	17.0 (8.0–81.0)	0.083
AST (U/L)	18.0 (11.4–44.6)	17.0 (13.0–56.0)	0.884
Hemoglobin (g/dL)	13.1 (9.8–15.6)	13.3 (9.2–17.2)	0.988
Hematocrit (%)	38.9 (14.4–47.8)	40.7 (29.0–55.0)	0.186
Ferritin (ng/mL)	19.9 (5.5–105.5)	14.6 (5.5–146.0)	0.927
Serum Iron (µg/dL)	78.0 (24.7–143.7)	70.0 (23.0–114.0)	0.378
Vitamin B12 (pg/mL)	241.0 (144.0–390.0)	330.0 (230.0–1645.0)	< 0.001
Folic Acid (ng/mL)	6.0 (3.7–10.3)	5.7 (2.3–10.0)	0.287
Total Calcium (mg/dL)	9.0 (7.8–10.0)	9.3 (8.5–10.5)	0.111
Sodium (mmol/L)	140.0 (136.0–142.0)	139.0 (136.0–144.0)	0.793
Potassium (mmol/L)	4.25 (3.70–5.20)	4.40 (3.73–5.30)	0.262

*FPG* Fasting Plasma Glucose, *HDL-C* High-Density Lipoprotein Cholesterol, *LDL-C* Low-Density Lipoprotein Cholesterol, *HOMA-IR* Homeostatic Model Assessment of Insulin Resistance, *ALT* Alanine Aminotransferase, *AST* Aspartate Aminotransferase

*Wilcoxon signed-rank test

**Table 7:** Comparison of dietary intake variables between the preoperative and postoperative year 8

Variable	Preoperative median (min–max)	Postoperative median (min–max)	p*
Energy (kcal)	1855.9 (997.0–3115.8)	1225.3 (844.1–2466.9)	0.001
Protein (g)	80.4 (44.5–151.5)	57.8 (32.8–1076.2)	0.274
Protein (%)	16.3 (10.7–27.7)	16.0 (12.0–90.8)	0.677
Fat (g)	146.3 (82.3–410.2)	55.5 (15.0–112.6)	< 0.001
Fat (%)	41.0 (28.3–61.0)	41.0 (30.0–70.2)	0.848
Carbohydrate (g)	85.0 (30.7–268.2)	116.4 (34.0–268.2)	0.122
Carbohydrate (%)	38.3 (16.7–50.3)	44.0 (24.0–143.8)	0.017
Dietary fiber (g)	18.0 (8.7–27.0)	13.4 (6.3–46.0)	0.590
Saturated fat (g)	30.9 (12.7–53.7)	19.4 (9.3–51.6)	0.007

*Wilcoxon Signed-Rank Test

**Table 8:** Comparison of macronutrient intake at postoperative year 8 according to RWG status

Variable	RWG > 30% median (min–max)	RWG < 30% median (min–max)	p*
Energy (kcal)	1638.80 (907.87–2230.50)	1284.96 (911.59–1598.57)	0.045
Protein (g)	65.34 (38.76–80.31)	55.15 (32.75–90.78)	0.166
Protein (%)	15.00 (12.00–33.00)	16.00 (13.00–26.00)	0.306
Fat (g)	70.30 (43.42–97.44)	60.15 (43.82–74.19)	0.166
Fat (%)	38.67 (30.00–47.00)	42.00 (39.00–50.00)	0.060
Carbohydrate (g)	189.53 (51.98–262.58)	116.36 (70.54–178.16)	0.052
Carbohydrate (%)	44.00 (24.00–58.00)	40.00 (27.00–46.00)	0.116
Dietary fiber (g)	15.87 (6.33–31.36)	12.08 (6.33–27.38)	0.148
Saturated fat (g)	31.16 (14.26–41.30)	21.08 (14.26–31.99)	0.052

*Mann–Whitney U test (two-tailed)

**Table 9:** Comparison of eating habits and lifestyle behaviors according to RWG status at postoperative year 8 (*n* = 23)

Variable	Category	RWG > 30% *n* (%)	RWG < 30% *n* (%)	Total	p*
Main meal pattern	Morning + Evening	2 (18.2)	9 (81.8)	11	0.013
	Morning + Noon + Evening	8 (80.0)	2 (20.0)	10	
	Noon + Evening	1 (50.0)	1 (50.0)	2	
Snack consumption	Mid-morning	0 (0.0)	2 (100)	2	0.015
	Afternoon	0 (0.0)	4 (100)	4	
	Night	3 (60.0)	2 (40.0)	5	
	Afternoon + Night	7 (87.5)	1 (12.5)	8	
	Mixed	1 (33.3)	2 (66.7)	3	
Breakfast	Yes	10 (52.6)	9 (47.4)	19	0.590
	No	1 (25.0)	3 (75.0)	4	
Fast food	Yes	8 (61.5)	5 (38.5)	13	0.169
	No	3 (30.0)	7 (70.0)	10	
Eating speed	Fast	7 (70.0)	3 (30.0)	10	0.211
	Moderate	2 (33.3)	4 (66.7)	6	
Perception of adequate and balanced nutrition	Yes	4 (28.6)	10 (71.4)	14	0.031
	No	3 (100)	0 (0)	3	
	Unknown	4 (66.7)	2 (33.3)	6	
Adherence to solid–liquid rule	Yes	3 (25.0)	9 (75.0)	12	0.039
	No	8 (72.7)	3 (27.3)	11	
Daily step count	Very sedentary	4 (80.0)	1 (20.0)	5	0.731
	0–2500	2 (50.0)	2 (50.0)	4	
	2500–5000	2 (33.3)	4 (66.7)	6	
	5000–7500	2 (40.0)	3 (60.0)	5	
	7500–10,000	1 (50.0)	1 (50.0)	2	
	> 12,500	0 (0)	1 (100.0)	1	
Regular physical activity	Yes	0 (0)	3 (100)	3	0.217
	No	11 (55.0)	9 (45.0)	20	
Sleep duration	4–6 h	6 (75.0)	2 (25.0)	8	0.089
	6–8 h	5 (33.3)	10 (66.7)	15	

*Fisher / Chi-square

**Table 10:** Preoperative and postoperative dietary patterns in bariatric patients with RWG

Food item	Period	Daily	Weekly	Rarely	Never
Milk	Preop	27.3	9.1	18.2	45.5
	Postop	18.2	36.4	27.3	18.2
Yogurt	Preop	63.6	18.2	18.2	0.0
	Postop	54.5	36.4	9.1	0.0
Cheese	Preop	72.7	18.2	9.1	0.0
	Postop	63.6	27.3	9.1	0.0
Egg	Preop	36.4	54.4	9.1	0.0
	Postop	54.5	45.5	0.0	0.0
Meat	Preop	0.0	100.0	0.0	0.0
	Postop	0.0	90.9	9.1	0.0
Legumes	Preop	27.3	27.3	45.5	0.0
	Postop	0.0	81.8	18.2	0.0
Bread	Preop	90.9	9.1	0.0	0.0
	Postop	90.9	9.1	0.0	0.0
Pasta / Rice / Pastry	Preop	18.2	81.8	0.0	0.0
	Postop	27.3	72.7	0.0	0.0
Dough-based foods	Preop	0.0	72.7	27.3	0.0
	Postop	18.2	72.7	9.1	0.0
Vegetables	Preop	27.3	36.4	9.1	27.3
	Postop	18.2	81.8	0.0	0.0
Salad	Preop	81.8	18.2	0.0	0.0
	Postop	54.5	27.3	18.2	0.0
Fruit	Preop	54.5	45.5	0.0	0.0
	Postop	45.5	54.5	0.0	0.0
Carbonated drinks	Preop	9.1	18.2	54.5	18.2
	Postop	0.0	9.1	36.4	54.5
Alcohol	Preop	0.0	9.1	9.1	81.8
	Postop	0.0	9.1	9.1	81.8
Packaged fruit juice	Preop	0.0	9.1	54.5	36.4
	Postop	0.0	0.0	54.5	45.5
Milk-based desserts	Preop	0.0	72.7	27.3	0.0
	Postop	9.1	72.7	18.2	0.0
Syrup-based desserts	Preop	0.0	0.0	90.9	9.1
	Postop	0.0	18.2	81.8	0.0
Chocolate	Preop	27.3	45.5	27.3	0.0
	Postop	0.0	100.0	0.0	0.0
Biscuit / cracker	Preop	0.0	100.0	0.0	0.0
	Postop	0.0	100.0	0.0	0.0

Values are presented as percentages (%)

## Discussion

This study represents one of the limited long-term investigations simultaneously evaluating anthropometric, biochemical, and nutritional outcomes before SG and at the 8-year postoperative follow-up [27–30]. The findings suggest that the long-term value of SG may extend beyond weight reduction alone, with metabolic and nutritional indicators also contributing to the assessment of outcome sustainability. These results support the concept that postoperative outcomes may be better interpreted within a multidimensional framework rather than relying solely on weight-based metrics.

The literature reports heterogeneous outcomes regarding %EWL at postoperative year 8. Some studies have reported %EWL values ranging between 51% and 62%, whereas others have described values approaching 70% [4, 8, 9]. The %EWL observed in the present study (60.16%) is consistent with these findings.

However, recent literature emphasizes that %TWL should be considered the primary outcome measure, as it provides a more standardized and reproducible metric independent of ideal body weight definitions [12, 22, 25]. In the present study, %TWL calculated based on nadir weight was 34.64%, whereas %TWL calculated based on current body weight was 26.4%.

Studies with comparable follow-up durations [7, 9, 27, 31] have reported lower %TWL values (19–23.5%) than those observed in our cohort. Taken together, these findings suggest that some degree of attenuation in weight loss over time is an expected component of long-term outcomes following SG.

Although RWG was observed in a substantial proportion of patients, overall weight loss remained within the long-term ranges reported in the literature. Therefore, RWG should not be interpreted as treatment failure but rather as a common component of long-term weight trajectories following SG.

Importantly, distinguishing between weight regain from nadir and net long-term weight loss allows for a more accurate and clinically meaningful interpretation of surgical outcomes.

Furthermore, the variability reported in the literature suggests that long-term weight trajectories after SG are not uniform and may differ considerably between patients. This heterogeneity underscores the importance of evaluating factors beyond absolute weight loss, including nutritional adaptation, behavioral patterns, and metabolic responses that may influence long-term sustainability.

Reductions in waist circumference, waist-to-hip ratio, and body fat percentage were observed, suggesting a sustained effect of SG on central adiposity. The marked decrease in waist circumference from values corresponding to a high cardiometabolic risk category in the preoperative period to substantially lower postoperative levels suggests meaningful clinical improvement [32]. However, the fact that postoperative values did not fully reach the normal range highlights the need for continued long-term metabolic surveillance following SG.

Consistent with the existing literature, comparison of the preoperative period with postoperative year 8 demonstrated sustained improvements in glucose metabolism and lipid profile [9, 14, 30, 31, 33–35]. Reductions in fasting plasma glucose and HOMA-IR levels may reflect sustained improvement in insulin resistance, whereas increased HDL-cholesterol and decreased LDL-cholesterol suggest a favorable shift in cardiometabolic risk. Similar to previous reports, a meaningful reduction in the prevalence of associated medical problems such as obstructive sleep apnea and polycystic ovary syndrome (PCOS) was observed [9, 14, 30, 31, 35]. Improvement in PCOS is biologically plausible given the central role of hyperinsulinemia and insulin resistance in its pathogenesis. Reduced insulin resistance decreases ovarian androgen production, increases sex hormone–binding globulin, and supports restoration of ovulatory function. The metabolic changes observed after SG likely contribute to reversal of this endocrine cycle, explaining the reduction in PCOS prevalence as an expected long-term outcome [36].

One of the debated aspects of SG is its relationship with gastroesophageal reflux disease (GERD) [9, 14, 30, 34, 37]. In the present cohort, GERD prevalence decreased from 65.2% to 39.1%; however, this change did not reach statistical significance (*p* = 0.180). Given the ongoing debate regarding reflux outcomes after SG, this finding should be interpreted with caution. The reliance on physician-diagnosed GERD without objective confirmation (e.g., endoscopy or pH monitoring) further limits the interpretation of these results. Therefore, these findings do not support a causal inference that SG reduces GERD risk, and individual variability in postoperative reflux patterns should be considered.

According to IFSO consensus criteria, suboptimal clinical response is defined as a %TWL below 20%, whereas RWG is defined as regain of ≥ 30% of the weight initially lost [23]. When %TWL was calculated relative to nadir weight, no suboptimal clinical response was observed at postoperative year 8. However, when %TWL was calculated based on current body weight, 34.8% of patients remained below the 20% threshold, and RWG was observed in 52.2% of patients at the 8-year postoperative assessment. These findings should be interpreted in the context of overall long-term weight outcomes. Although RWG was observed, it should not be considered treatment failure, as substantial net weight loss was maintained. Due to the limited sample size, the study was not powered for multivariable analyses, and therefore potential predictors of RWG could not be evaluated using regression models.

When all patients were evaluated collectively, postoperative reductions in total energy, fat, and saturated fat intake were observed, accompanied by an increased proportional contribution of carbohydrates to total dietary intake. Although absolute carbohydrate intake increased, total energy intake decreased, reflecting a shift in macronutrient composition, particularly a substantial reduction in fat intake, which has a higher caloric density. This pattern suggests a relative increase in the contribution of carbohydrates to total energy intake rather than an overall increase in caloric consumption. Long-term nutritional recommendations from the ASMBS emphasize individualized energy prescriptions with a protein-focused macronutrient pattern and controlled carbohydrate intake [38].

In the present study, postoperative year-8 macronutrient intake differed according to RWG status. Patients experiencing RWG had higher daily energy intake compared with those with lower regain (*p* = 0.045). Similar findings have been reported by Alvarez et al. [39], who noted that patients with RWG after SG tended to consume higher levels of energy and fat than weight-maintaining peers. Essayli et al. [40] likewise emphasized a shift away from healthy dietary behaviors and increased caloric intake in patients experiencing RWG. Consistent with these observations, Iossa et al. [17] demonstrated that energy and macronutrient intake were higher in patients with RWG 7 years after SG compared with those who maintained weight reduction.

Although protein intake in the present cohort appeared to meet minimum recommended levels, its proportional contribution to total energy intake was relatively low, while carbohydrate and saturated fat intake approached the upper recommended limits. This pattern suggests an energy-dense dietary profile. Fiber intake remained below recommended levels in both groups, consistent with previous reports [41, 42].

Taken together, these findings suggest that RWG is less likely to be attributable to deficiency of a single macronutrient and more closely related to chronic positive energy balance and deterioration in dietary quality. Therefore, long-term follow-up after SG should extend beyond monitoring body weight alone and include regular assessment of energy intake and overall dietary patterns.

A difference was observed between meal patterns and RWG. Patients without RWG more commonly reported consuming two main meals (morning and evening), whereas those experiencing RWG tended to consume three main meals (morning, midday, and evening). Prior research similarly indicates that grazing behavior and uncontrolled eating patterns after SG are closely linked to RWG [10, 27, 37, 43]. These findings suggests that reduced meal frequency and a more temporally structured eating pattern may offer potential advantages for long-term weight maintenance. In addition, the higher prevalence of late-night snacking among patients with RWG may reflect uncontrolled energy intake, preference for energy-dense foods, and eating patterns misaligned with circadian rhythms. From this perspective, time-restricted eating models aligned with circadian biology may hold promise for long-term weight maintenance after bariatric surgery; however, prospective studies are needed to clarify causal relationships in this population. However, given the exploratory nature of the study, this observation should be interpreted cautiously.

From a dietary pattern standpoint, patients with RWG demonstrated persistently higher intake of bread and refined carbohydrates relative to the preoperative period, reduced consumption of vegetables and fruits, and continuation of energy-dense snacks. These behaviors are consistent with previously described behavioral profiles associated with postoperative RWG [5, 44, 45]. In contrast, the observed reduction in carbonated beverage consumption represents a partially divergent finding compared with some prior reports [33, 46]. Higher rates of RWG among patients who perceived their diet as inadequate or who did not adhere to the solid–liquid separation rule further support the importance of compliance with recommended postoperative eating behaviors for long-term weight maintenance. Previous reports similarly note that a substantial proportion of patients experiencing RWG demonstrate poor adherence to solid–liquid separation and reduced physical activity levels after MBS [27]. Interventions targeting unhealthy dietary behaviors and physical inactivity have been shown to reduce RWG risk and behavioral or lifestyle interventions appear to provide partial benefit in long-term postoperative weight management [37, 47–50]. Although no statistically significant association was observed between daily step count or regular physical activity and RWG in the present study, the absence of pronounced RWG among patients engaging in consistent activity may reflect a trend that could not be confirmed due to limited sample size. The observation of greater RWG among patients with shorter sleep duration further highlights the contribution of behavioral and lifestyle factors to long-term weight regulation.

A major strength of this study is the comprehensive evaluation of anthropometric, biochemical, and dietary parameters, along with associated medical conditions, in a long-term SG cohort, an area in which the literature remains limited. In addition, the comparative assessment of lifestyle and dietary behaviors between patients with and without RWG provides clinically relevant insight into long-term postoperative weight management.

This study has several limitations that should be considered. First, the relatively small sample size limits statistical power and restricts the ability to perform multivariable analyses; therefore, the findings should be considered exploratory. Second, the absence of structured year-by-year follow-up data limits the ability to evaluate temporal changes across the postoperative period. In addition, multiple statistical comparisons were performed across a wide range of outcomes without formal correction for multiple testing, which may increase the risk of Type I error (false-positive findings). Accordingly, findings, particularly those with p-values close to the significance threshold, should be interpreted with caution. Furthermore, although baseline characteristics were comparable between participants and non-participants, the possibility of attrition bias due to loss to follow-up cannot be entirely excluded. These limitations may affect the generalizability of the results and should be considered when interpreting the findings, although the study still provides valuable long-term follow-up data.

## Conclusion

Outcomes at postoperative year 8 after SG suggest that the impact of surgery extends beyond initial weight loss and involves sustained metabolic and behavioral adaptation. Although weight reduction was preserved in a substantial proportion of patients, RWG was observed in a subset of patients and was related to dietary patterns and lifestyle behaviors. Given the exploratory nature of the study and the limited sample size, these findings should be interpreted with caution. Larger prospective studies are needed to further clarify long-term weight trajectories and to support effective strategies for sustained weight management.

## Data Availability

Due to privacy and ethical restrictions, the data are not publicly available but may be available from the corresponding author in anonymized form upon reasonable request.
